# Acceptability of a digital program for training community health workers in the early detection and referral of schizophrenia in rural India: A mixed-methods pilot study

**DOI:** 10.1016/j.ssmmh.2026.100635

**Published:** 2026-04-24

**Authors:** John A. Naslund, Vidhi Tyagi, Deepak Tugnawat, Anant Bhan, Azaz Khan

**Affiliations:** a Department of Global Health and Social Medicine, Harvard Medical School, Boston, MA, United States; b Sangath, Bhopal, India

**Keywords:** Schizophrenia, Task-sharing, Global mental health, India, Community health worker, Rural, Digital technology

## Abstract

**Objective:**

Delays in detecting and treating schizophrenia result in elevated disability for patients. This mixed-methods pilot study evaluated the feasibility and acceptability of a digital program for training community health workers on early detection and referral of schizophrenia in primary care in rural India.

**Methods:**

Using convenience sampling, female community health workers, called ASHAs, enrolled in a large depression care study were invited to complete a supplemental digital training on identifying and responding to schizophrenia. Acceptability of the training was assessed through qualitative focus group discussions and satisfaction questionnaires. Framework analysis was used to code participants’ feedback about the training and generate representative themes. Pre-post changes in knowledge about schizophrenia were also explored.

**Results:**

In total, N = 20 ASHAs completed the training and participated in focus group discussions. Qualitative data revealed that participants found the training helpful, while they reflected on their limited prior understanding of schizophrenia. Participants emphasized that they had previously observed symptoms of schizophrenia in their communities, yet they did not realize that schizophrenia is a mental health condition that can be treated, and that rehabilitation efforts can be successful. Participants recommended conducting the training with other ASHAs to expand access to this information. Participants’ reported high satisfaction with the training, though showed no significant pre-post improvement in knowledge about schizophrenia.

**Conclusion:**

A scalable digital training may help equip community health workers to recognize schizophrenia and link people to care. Further evaluation is needed to assess effects on patient outcomes, and integration of schizophrenia care into primary care.

## 1. Introduction

Significant trained human resource constraints represent a key barrier to the early detection and initiation of care for persons living with schizophrenia in rural settings (Lora et al., 2012; Peritogiannis and Samakouri, 2021). These challenges are further exacerbated in low- and middle-income countries (LMICs) such as India (Gautham et al., 2020), where delays in seeking care and starting treatment often exceed 2 years (Mishra et al., 2021). These delays contribute to higher morbidity and mortality among individuals living with schizophrenia, and result in detrimental consequences to their functioning and increased burden and psychological distress for family caregivers (Paquin-Goulet et al., 2023; Thirthalli et al., 2011). Treatment seeking is often delayed because of limited awareness about schizophrenia and the associated symptoms, as well as stigma, representing concerns that are prominent in rural India (Dutta et al., 2019). Innovative strategies are needed to raise awareness and strengthen the community workforce for early detection and care.

Research shows the effectiveness of psychosocial interventions, including social skills training, cognitive behavioral therapy, or family psychoeducation, when combined with psychiatric care on improving symptom remission, functioning, illness self-management, and preventing relapse among individuals living with schizophrenia (Bighelli et al., 2021). These proven interventions can be adapted to train community health workers (CHWs) in low-resource settings. Referred to as task-sharing, CHWs without specialized mental health training can be supported in the effective delivery of these interventions for schizophrenia care in LMICs (Asher et al., 2017; Chatterjee et al., 2014). However, task-shared care for schizophrenia has largely focused on management of chronic schizophrenia, promoting functioning and preventing relapse (Asher et al., 2022; Chatterjee et al., 2014), with less emphasis on offering general skills training in identifying symptoms and supporting individuals and their families to seek treatment, while affording continued follow up care at the community level to promote adherence.

This pilot study aimed to address this gap by training CHWs, called Accredited Social Health Activists (ASHAs), in the detection and referral of individuals living with schizophrenia in community settings in rural India. Specifically, ASHAs are women health workers deployed within India’s National Health Mission, and they work at the community levels in rural and underserved settings (Scott et al., 2019). ASHAs play a critical role in delivering rural primary care services and represent a trusted resource within the communities where they live and work (Scott et al., 2019). ASHAs typically make home visits, meeting community members where they are, and offer a range of preventative services, maternal and child health care, and screening and follow up for chronic conditions. There has been growing emphasis on involving ASHAs in supporting mental health services (Nirisha et al., 2023; Varshney et al., 2022), because they are ideally positioned for community screening and supporting early identification and ongoing support for individuals living with schizophrenia (Sivakumar et al., 2022). Given their close connection with the community, they could help address challenges and barriers to seeking care among patients and caregivers such as stigma. It is necessary to explore the acceptability of a brief training and whether it could be integrated into their routine work to support acquisition of new skills and knowledge about schizophrenia.

Digital training programs, accessible through a smartphone application, offer a potentially appealing approach for reaching ASHAs and scaling up access to instructional content without requiring attendance in classroom-based or residential trainings (Brahmbhatt et al., 2024; Muke et al., 2019). The more costly in-person methods of instruction, typically taking place in government training facilities, remain the standard of practice for CHW training in India (Manjunatha et al., 2018). By leveraging increasingly available smartphone ownership and mobile internet coverage, ASHAs could complete a brief mental health training program during their field work, such as while traveling long distances by bus between villages, or while at home at their own leisure. With access to training content via a smartphone app, ASHAs could revisit materials at any time (Muke et al., 2019). Therefore, this study addresses a key gap by evaluating whether digital, asynchronous training is feasible and acceptable for ASHAs and can strengthen their knowledge of schizophrenia to support early detection and referral in rural communities. This study forms part of a broader program of research employing technology for training ASHAs in the delivery of a psychosocial intervention for depression that has been successfully scaled within the same region of India (Agrawal et al., 2025; Tugnawat et al., 2024).

Our team designed a brief digital training program drawing from a proven psychosocial intervention for community-based rehabilitation in schizophrenia previously developed and tested across different settings in India (Balaji et al., 2012; Chatterjee et al., 2014). A step-wise approach was employed for designing the digital training, with close engagement of ASHAs throughout this process to ensure cultural and contextual relevance for end-users (Tyagi et al., 2023). Specifically, this pilot study aimed to evaluate the acceptability of the digital training program among ASHAs based on their feedback and insights collected through qualitative focus group discussions (FGDs). We also explored the preliminary impact of the digital training on improving learning outcomes, measured as ASHAs’ change in knowledge about schizophrenia. Our goal was to gather feedback from ASHAs to inform further modifications to the training content and to guide efforts to build workforce capacity in rural settings and support the integration of schizophrenia care into primary care services in India.

## 2. Methods

### 2.1. Study setting and participants

This study was conducted in Sehore, a rural district of Madhya Pradesh, India. Participants included ASHAs and ASHA Facilitators, who are all women, recruited from primary healthcare facilities within India’s National Health Mission. Each ASHA covers a population of about 1000 and receives a base monthly remuneration and performance-based and service-based incentives for services including facilitating immunization, timely perinatal care access, facility-based childbirth and family planning (National Health Mission, 2023). Each ASHA Facilitator provides supportive supervision and on-site assistance for a group of 15-20 ASHAs and serves as the link between the ASHAs and the facilities for supporting care delivery at the community-level. ASHAs and ASHA Facilitators do not currently receive specialized training in mental health care, and do not receive any training about schizophrenia care. Eligible ASHAs or ASHA Facilitators were age ≥18 years and had minimum of 8th grade education to ensure sufficient reading level to complete the training and operate the smartphone device. ASHAs with significant speech, sight, or hearing impairment, or who are illiterate, were excluded. Eligible ASHAs were also enrolled in a larger study focused on evaluating a digital training program on depression care (Naslund et al., 2021; Tugnawat et al., 2024). We targeted this larger cohort (N = 339 ASHAs) to reach our convenience sample for this pilot study. Therefore, ASHAs entered this study with prior exposure to the digital training platform and content related to mental health and depression care, though no prior training about schizophrenia. All study procedures were approved by Institutional Review Boards at Sangath, India (Number: JN_2019_64) and Harvard Medical School, USA (Number: IRB20-1164).

### 2.2. Digital training program

Participants completed a digital training consisting of 6 modules covering topics such as: learning about schizophrenia, the associated symptoms and phases of illness, misconceptions, stigma, and available treatments; understanding the impact of schizophrenia on disability and functioning, and recognizing the importance of community-based rehabilitation; understanding how to identify possible cases of schizophrenia in the community; the role of family, offering psychosocial support, and engaging family in the care process; and making a referral to psychiatric care through the district mental health program. The training content was adapted from evidence-based psychosocial rehabilitation programs for schizophrenia, including the COPSI (Community Care for People with Schizophrenia in India) program in India (Chatterjee et al., 2014) and the RISE (Rehabilitation for People with Schizophrenia in Ethiopia) program in Ethiopia (Asher et al., 2022). Together, these programs offer evidence-based psychosocial rehabilitation content that was simplified and tailored to the needs of ASHAs (Chatterjee et al., 2014). Further, the training content was translated into Hindi and reviewed by ASHAs to provide feedback about the language and terms. The digital training consisted of short videos, developed in collaboration with a local video production company and filmed in community settings to reflect locations familiar and relatable to ASHAs. Graphics and images depicting care for schizophrenia were used to supplement the video-based content, and the training was accompanied by a printed manual. The step-wise process for developing the digital training program is described previously (Tyagi et al., 2023), and involved engagement of clinicians, persons with lived experience, and ASHAs.

### 2.3. Procedures

To evaluate the acceptability of the digital training program, we used a convenience sampling approach where we invited 20 ASHAs enrolled in a larger study with over 300 participants focused on training and scaling up depression care (Naslund et al., 2021, 2022; Tugnawat et al., 2024). We stopped recruitment after reaching our target of N = 20 ASHAs for this pilot study. This sample size was feasible to recruit in the project timeframe, while allowing emphasis on collection of both qualitative and quantitative data on program acceptability, and is consistent with the design of prior mixed methods pilot studies (Aschbrenner et al., 2022; Teresi et al., 2022). The ASHAs entered this study with some prior exposure to content related to mental health and depression, but no prior training about schizophrenia. Interested ASHAs were invited to an information session about the study procedures and the training program about schizophrenia. For ASHAs who consented, they completed a baseline knowledge questionnaire about schizophrenia and joined an in-person orientation about accessing the digital training content. Participants had up to 4 weeks to complete the training, after which they were invited to join qualitative FGDs and complete an endline assessment. Throughout the study, experienced counselors on the research team were available to offer support to participants in the event they experienced distress from accessing the training content. We employed multiple measures and a combination of qualitative and quantitative data to capture acceptability, conceptualized using Proctor et al. (2011) framework as participants’ perceptions of the training’s content, usability, and perceived value (Proctor et al., 2011). Acceptability was assessed using qualitative feedback from FGDs, satisfaction questionnaire scores, and program completion rates.

### 2.4. Quantitative data collection

Quantitative measures supplemented the qualitative FGDs. This included a pre-post knowledge assessment designed for this study to explore initial program impact on learning outcomes reflected as change in their: general knowledge about schizophrenia, and associated symptoms; approaches for detection and referral of schizophrenia in primary care and community settings; and methods for communicating with and offering support to family caregivers of persons living with schizophrenia. The self-report knowledge assessment has 26-items consisting of short 1-2 sentence questions followed by 4 multiple-choice response options. For each question, there is only one correct response, and partial credit is not awarded. Scores range from 0 to 26, with higher scores indicating greater knowledge. This knowledge assessment was not validated or tested ahead of this study, and therefore, was included as an exploratory measure to supplement the qualitative findings on the training feasibility and acceptability as the sample size was underpowered to detect pre-post changes. Design of the knowledge assessment and translation for the setting in India modeled an approach our team employed previously for assessing knowledge to deliver a brief psychosocial intervention for depression (Joshi et al., 2022; Restivo et al., 2020).

We also collected participants’ satisfaction with the training using a 26-item questionnaire adapted from the MUSIC^®^ model, a measure of motivation and engagement in training programs (Jones, 2009, 2010, 2017). This measure covers feasibility, acceptability, adoption, and appropriateness of the training program, and a Hindi version of the questionnaire was used in a prior study (Muke et al., 2020). The items are rated on a 6-point Likert scale and grouped into domains reflecting participants’ motivation and engagement in online education programs, with higher scores indicating greater satisfaction (Jones, 2017).

### 2.5. Statistical analysis

Descriptive statistics were used to summarize participant demographic characteristics. For the pre-post changes in the knowledge scores, we found that the data were not normally distributed. Therefore, we used the Wilcoxon signed rank test as an alternative to the *t*-test. The satisfaction questionnaire results were summarized using descriptive statistics. All statistical analyses were completed using Stata Version 18.0, and p-values less than 0.05 were considered significant.

### 2.6. Qualitative data collection

Two members of the research team used a semi-structured interview guide to facilitate three FGDs in Hindi with 6-8 participants in each. The interview guide was developed based on our study objectives focused on understanding acceptability of the training program, and informed by Proctor et al.’s (2011) implementation outcome framework (Proctor et al., 2011), covering topics such as satisfaction, usability, and perceived utility/usefulness. We gathered input from our prior formative work related to the development of the training (Tyagi et al., 2023) as well as our prior work with ASHAs in the same study setting (Muke et al., 2019), and allowed for iterative refinement with the research team to ensure key aspects of feasibility and acceptability were captured in the guide and that the questions were relevant for the context and target participant group. The FGDs were audio-recorded, transcribed verbatim and translated into English for thematic analysis and presentation of the findings. Two experienced members of the research team listened to the audio-recordings and reviewed the quality of the transcriptions. Two additional members of the research team and the lead author reviewed the transcripts to become familiar with the data. This process was used to document initial impressions of the data and define topic categories to develop a codebook to facilitate the framework analysis.

### 2.7. Thematic analysis

We used framework analysis applying a deductive coding approach (Gale et al., 2013), appropriate for pilot studies in which analysis is informed by predefined research questions and specific implementation outcomes (i.e., focus on feasibility and acceptability) (Aschbrenner et al., 2022; O’Cathain et al., 2015). Given our main objective was to understand the feasibility and acceptability of the digital training program, in planning for conducting a future large-scale evaluation, this analytic approach allowed us to draw from participants’ feedback, insights, and recommendations for improving the training content. The qualitative data offered the opportunity to expand upon the quantitative findings, such as participants’ satisfaction ratings with the training. Two members of the research team independently reviewed the transcripts, using line-by-line coding following an a priori codebook grounded in conceptual definitions of program feasibility and acceptability (Proctor et al., 2011). They met with the lead author to review the codes and discuss observations in the data. This process involved grouping codes into overarching themes and reaching consensus through iterative discussions. We shared the themes, codes, and representative quotes with the research team, including members experienced in community-based rehabilitation and training design. This process allowed for the collection of additional feedback and integration of suggestions for interpretation of the findings.

## 3. Results

### 3.1. Participant characteristics

All 20 ASHAs enrolled in this study completed the digital training program, including all 6 modules, within the 4-week timeframe provided and then participated in the FGDs. Participants ranged in age from 26 to 45 years. Half the participants (n = 10; 50%) had 8–10th grade education, and over half had at least 10 years of work experience (n = 11; 55%). Table 1 summarizes participant demographic characteristics.

### 3.2. Knowledge assessment scores

A Wilcoxon signed-rank test revealed no significant difference in the pre- and post-knowledge assessment scores (Z = −1.158, p = 0.247), with a median score of 11.32 (mean = 11.53; SD = 2.10) before the training and 12.16 (mean = 12.66; SD = 2.61) after the training.

### 3.3. Satisfaction questionnaire

The satisfaction scores were generally favorable for most items, reflecting participants positive perceptions and experiences with the training program and content. Participant responses to the satisfaction questionnaire are summarized in Table 2.

### 3.4. Qualitative findings

Thematic analysis of the FGDs revealed the following overarching themes aligned with the semi-structured interview guide: acceptability of the digital training; limited prior understanding and misconceptions about schizophrenia; and knowledge and skills gained from the training (see Table 3). These themes and illustrative quotes are summarized in the sections that follow.

#### 3.4.1. Theme 1: acceptability of the digital training

Participants expressed an appreciation for the training content, and indicated that they found it easy to understand, commenting on the use of language that was relatable and familiar to them. One ASHA commented:

“*The content was also good, meaning the language was good and easy to understand. There was nothing different, it was just our mother tongue Hindi which we can understand, neither was there English in it nor was the video fast* …” (FGD1).

Participants shared positive perceptions about the graphics and presentation of the content. This included comments about the value of using videos, such as role-plays, and using images or graphics to reinforce the learning objectives and concepts. One ASHA mentioned:

*“After that, madam showed role play through video and also through pictures, so that was also good, it was shown through pictures.”* (FGD2)

Several participants also commented on the ease of navigating the digital platform from their phones, highlighting the value of being able to revisit content and review materials as often as they would like. One ASHA expressed her appreciation with this feature:

*“Yes, we can do it, watch that video once again, then watch it twice, you can watch it again and again if you are not able to understand.”* (FGD2)

One participant reflected on the differences between digital training compared to conventional in-person instruction. This participant highlighted that in face-to-face training there are fewer opportunities to ask questions, because it is only possible to ask a question once or twice if you can think of it during class, whereas the digital training offers the advantage of being able to review and revisit the content as often as needed:

*“In face to face we can ask question once, Sir will tell but we can ask question only when it comes to mind, but whatever is on mobile, we can try it again and again from start to end, we can go wherever we want, we can do it by doing anything. In face to face, we can ask question once or twice, we can explain it, we cannot explain it again and again. … the mobile one is good, if you are not able to understand in one go, then watch the video, […] according to your convenience, whenever you get time, you can do it.”* (FGD1)

Some participants commented on challenges they encountered during the training, such as finding content unclear, or difficulty navigating activities or questions. This was reflected in the following comment:

*“I could not understand which one to click and which one not to, is its answer correct or not […], they all looked similar, so sometimes it was difficult to see.”* (FGD2)

Similar challenges were mentioned by another participant who described finding it difficult to respond to questions embedded in the digital content:

*“… if I don’t do it then it gets mixed up in between, that’s why, let’s say if one of the two questions is left out then there is confusion and sometimes it does not come, because of this sometimes questions are left out, how were they marked, yes I am thinking that I am doing it well but sometimes I miss them.”* (FGD3)

#### 3.4.2. Theme 2: limited prior understanding and misconceptions about schizophrenia

Participants reflected on their understanding and perceptions about schizophrenia prior to the training. Interestingly, nearly all participants expressed that prior to the training, they did not know that schizophrenia is a mental health condition and that it can be treated. One ASHA commented, “*I have neither heard the word nor heard about the disease nor seen its symptoms*” (FGD1). This was expressed by another participant who also had not heard of the term ‘schizophrenia’ previously:

*“Madam, I never thought that this disease, this disease with this name, I never thought that there is a disease with this name.”* (FGD3).

Another ASHA described an instance where she encountered a young man with these symptoms in her village, and did not realize that it was an illness that could be treated:

*“When I saw him, I felt a little strange that maybe some of the things that we talk about throughout the day get stuck in his mind and when I go to sleep at night, I think it is possible that he might be hearing some such voices or seeing something and I did not pay much attention to it because I never knew that such a disease also exists.”* (FGD1).

Participants described instances when symptoms of schizophrenia are observed in their villages, it is typically associated with witchcraft or black magic:

*“In the village, people used to say that he has gone mad, he has lost his mind and all such things. Yes, they say that everyone in the village used to say that it means that someone has done black magic on that boy, it seems as if he is possessed by a ghost. That is why he keeps coming again and again. People in the village keep saying this but now after taking this training, I have come to know that this is a disease of mental health as we say.”* (FGD2).

This was also reflected by another ASHA, who mentioned that when such symptoms are observed, then the afflicted individual would be taken to a village elder or faith leader, and would be considered ‘mad’, a label that would persist for the rest of the individual’s life. Such insights further highlight that there was no recognition of this as a medical condition:

*“… they take them to babas etc. and start treatment and finally declare them mad, so they become whatever people say they are mad for the rest of their life because even we did not know before that there is a cure for it. But after training we came to know that this is a disease.”* (FGD1).

Participants also talked about the fears and misconceptions about people who they had seen with symptoms of schizophrenia in their communities. This included being afraid to go near these individuals:

*“I am afraid to go near him, I mean that he might cause some harm, I don’t know what comes into his mind then? Because of that people start getting scared.”* (FGD1).

Another participant commented on how this condition and symptoms have been attributed to an individual’s own will, suggesting that the individual chooses to act this way:

*“After seeing this, I felt that their mental balance is not right because you can pay a little attention to this thing, but those people do not understand their own words. No, we have not paid much attention to them. Many people think that they do things according to their own will.”* (FGD2).

#### 3.4.3. Theme 3: knowledge and skills gained from the training

Participants also described skills and knowledge they learned from the training. This included gaining a better understanding of the different symptoms, that these symptoms are attributed to a mental health condition called schizophrenia, and that there are treatment options available. Several ASHAs commented on the importance of recognizing symptoms of schizophrenia in their communities:

*“After the training we felt that by doing training we should understand the power of recognizing the symptoms of disease and even think that if a person’s life can be saved after this training then he can also be saved from going mad, we can also save him from causing other harm, and the other problems in this are such that how can we handle some things, we should see through video how can we handle him.”* (FGD1).

Another participant commented that she feels like she would be able to explain the condition to the family members of an affected individual:

*“First of all, we will ask all these questions and if he is not able to understand, then we will talk to his family members. We will explain to them that there is no black magic because nowadays in villages people think that this is black magic or ‘maya’(supernatural power) or some deity, if they are affected by it then it is not a ghost or spirit, then we will explain to them that it is a disease and it can be treated, we will give them advice and also take them to the nearest health center in the nearby area, we will give them this advice […], we will have to talk to the family members if they are not in a position to understand it.”* (FGD2).

Several ASHAs also described scenarios where they could apply this knowledge in their work and with people they meet in their villages. For instance, one ASHA commented that she would now be able to identify these symptoms and understand how to respond appropriately:

*“The best thing is that we have knowledge about everything, but we did not know what is the disease of schizophrenia, when we took the training then it felt good that yes, what is schizophrenia called, now we got training for it, […], so when we go to every house, we meet all kinds of people in every village, so anyway it will become very easy to recognize it, well, how do we not understand it. Earlier we did not understand it well, but now that we have come to know about it, so now we know what can happen.”* (FGD2).

Participants also mentioned finding that the content from the training would help them in interacting with and supporting family members of affected individuals, and how they would approach these scenarios in their villages:

*“Now we will first give this advice to the family members. We will ask since when this has been happening and try again. Yes, sometimes what does the patient do, he starts laughing, starts talking to himself, so we will advise that all this can be cured during treatment.”* (FGD1).

This was similarly reflected by another ASHA, who highlighted the importance of listening to the patient, and that the family would also listen to her if she explained that this is an illness:

*“The patient will understand this way that if someone listens, he will listen, otherwise he will start getting angry, then the family members will have to explain. Yes, the family members will explain, I will explain a little, if they hear it from me, they will listen and the family will definitely listen.”* (FGD3).

The same ASHA continued, describing how she would explain the illness to family members, while asking questions about how long the symptoms have been occurring and whether they have consulted a doctor:

*“You have to ask them that is there something like this in the mind, like did you consult a doctor? Did you go and consult someone, are you already undergoing treatment, does it mean that it has been like this since childhood […], so is it something in the brain that is a disease, that means it happened to you in the middle, so you have to show it to them that they don’t listen, like first of all, no one listens. At home, mother will surely listen.”* (FGD3).

Several participants mentioned that they now had a better understanding of the benefits that would be available to an individual living with schizophrenia, and the possibility of securing a disability certificate from the government:

*“It has also been explained about the disability certificate, what are the documents required for it, and from where will it be made. Well, one thing in this is that a patient suffering from schizophrenia … should get regular treatment, then he will be cured, that means he will be able to do his job like he used to do. The one who is studying can also study further. This is a temporary disease. Yes, after taking medicine and treatment, the condition will become normal and he will be cured.”* (FGD2).

Lastly, many participants emphasized the importance of the knowledge gained, indicating that they could help get an individual into treatment and that they could help show a patient the *“right path”* rather than relying on superstitions at the village level:

*“… we want the maximum number of people to know about it, that schizophrenia is a disease because nowadays most of the people live with superstitions. If he gets the right treatment then it is our job to show him the right path; if he gets the right path then instead of going through exorcism, he can get cured quickly with the treatment of doctor.”* (FGD1).

## 4. Discussion

This pilot study highlights the acceptability of a digital program for introducing ASHAs to content about schizophrenia, how to identify symptoms in the community, communicate with and offer support to family members, and refer persons living with schizophrenia to specialty care. Acceptability of the training was reflected by full completion among all participants, favorable satisfaction ratings, and positive feedback shared during the FGDs. While our knowledge outcome did not show statistically significant improvement, when drawing from our qualitative findings, participants reported gaining valuable knowledge about schizophrenia. For instance, participants indicated that they were not aware of a mental health condition called ‘schizophrenia’ before the training, and while they had encountered symptoms of schizophrenia previously, they were unaware that this could be treated and that they could support individuals living with schizophrenia and their caregivers. Participants’ comments illustrate the importance of training communitylevel providers about schizophrenia and making this information more widely available to dispel misconceptions and raise awareness.

A strength with our study is that the digital training was intentionally designed as a brief self-paced program accessible from a smartphone that participants could complete at their own convenience. ASHAs are overburdened in their work and face increasing workplace demands (Mitchell et al., 2024; Shrivastava et al., 2023), emphasizing the need for skill-building without creating additional burden. There are opportunities to scale up this digital training considering the few required resources, and in recent years, access to smartphones among ASHAs has increased substantially across India (Vasanthan et al., 2024). This is partly due to the accelerated adoption of digital technology during the COVID-19 pandemic as a necessity for carrying out basic activities (Dash et al., 2021), along with the rollout of national programs and initiatives to equip ASHAs with smartphones to facilitate their work (Bashingwa et al., 2021; Srinidhi et al., 2021). Our study draws from recent examples demonstrating the role of digital technology in ensuring sustained access to community-based rehabilitation services for schizophrenia within the context of the COVID-19 pandemic in settings in Karnataka, India (Sivakumar et al., 2023; Sivakumar et al., 2022). Further, increasing access to smartphone technology has also benefited from improved connectivity, even in rural areas. This contrasts our prior formative research conducted before the pandemic, where we observed many technical challenges with low bandwidth for using a smartphone-based training with ASHAs (Muke et al., 2019). Interestingly, we did not observe similar challenges in the current study. The gap of a few years between the two studies reflects trends in improved connectivity and increasing digital literacy among ASHAs.

Our study supports task-sharing efforts in rural India, and advances existing studies aimed at improving ASHAs’ attitudes and understanding of schizophrenia (James et al., 2019). Our study expands on this prior work by demonstrating the potential for a scalable digital program to facilitate broad access to training content surrounding schizophrenia care. Despite strong evidence for task-sharing and psychosocial interventions in schizophrenia care, few initiatives have focused on equipping CHWs in the government health system to identify and refer cases early at the community level (Brooke-Sumner et al., 2015; Farooq et al., 2024). A recent program in Karnataka, India focused on training ASHAs in the delivery of community-based rehabilitation for persons with severe mental disorders as a complement to usual psychiatric care (Sivakumar et al., 2020). Another study in Karnataka demonstrated the potential for community-based psychosocial rehabilitation to reduce out-of-pocket expenses for individuals living with schizophrenia (Sivakumar et al., 2019), thereby highlighting the need to consider the cost implications and potential health system and broader societal savings from training ASHAs. There may also be unique advantages to training ASHAs relative to other cadres of health workers, given they are a trusted resource within their villages and like many other community-based providers, are typically from the communities where they work, and may be ideally positioned to speak to the family members of individuals living with schizophrenia (Carrara et al., 2023). Prior studies have demonstrated that stigma at the family level can result in significant delays in help-seeking and initiating care for individuals living with schizophrenia (Koschorke et al., 2017; Mishra et al., 2021). By training ASHAs to recognize symptoms of schizophrenia and understand the misconceptions about the illness, they may be able to directly address barriers such as stigma within their communities and encourage early access to care. For instance, ASHAs may be able to talk with family members, offer explanations for the unusual behaviors or symptoms of a loved one, and offer reassurances while also dispelling myths or superstitions. Training ASHAs may help to embed this capacity at the community-level, and overcome previously reported challenges with achieving sustainability of schizophrenia care in primary care (Thara et al., 2008).

### 4.1. Limitations

Several limitations warrant consideration. The small convenience sample recruited from a cohort who had previously completed a digital depression training limits generalizability. Participants had prior exposure to the digital training platform and mental health content, which may not reflect ASHAs without such experience and may indicate higher digital literacy or interest in mental health in the current study. It is noteworthy that none of the participants were aware of schizophrenia prior to this training. Recruitment involved sharing information about this pilot study to a larger cohort of ASHAs enrolled in a depression care training program, and was closed once the target sample size of 20 ASHAs was reached. As such, refusals or reasons for non-participation were not tracked, and non-response does not necessarily reflect an explicit decision to decline participation. That said, for future training efforts focused on schizophrenia, it is unlikely that all ASHAs would be trained given the relatively low prevalence of the condition. Instead, an approach that targets ASHAs with prior knowledge in common mental health conditions may be ideal and positions schizophrenia care as an advanced training. All participating ASHAs joined the qualitative FGDs and reported high satisfaction with the training program. Given these ASHAs were already engaged in a larger research study, they were familiar with the research team and may have been more motivated and inclined toward favorable responses. This introduces potential desirability bias and limits generalizability to the broader ASHA workforce. Additionally, the knowledge assessment measure was exploratory given that this study was not sufficiently powered to detect pre-post changes, and the measure was not validated ahead of use in this study. The same knowledge measure was used before and after the training, which could influence scores. Therefore, the knowledge scores should be interpreted with caution.

Another important limitation is that we were unable to assess whether participants applied what they learned in the communities where they work. This highlights a key area for future research, to determine how this brief training may influence how ASHAs respond to cases of schizophrenia within their communities, and whether there is an impact on case detection, referral, and treatment initiation in rural settings. Further, we acknowledge that while the training was acceptable to participants, acceptability alone does not ensure scalability or long-term sustainability, especially in rural settings where supervision, digital infrastructure, and ongoing support may be limited (Singla et al., 2024). Future research will be needed with long-term follow up at the community-level to determine whether training ASHAs in these skills and knowledge can support early detection of schizophrenia and address delays in initiating care. We also acknowledge that our training program alone will likely not be sufficient to develop the skills and competencies required for effectively supporting individuals living with schizophrenia in community settings (Valenstein-Mah et al., 2020). Prior research shows that while education can improve knowledge, it does not always lead to changes in clinical practice (McCluskey and Lovarini, 2005). This underscores the importance of practice-based learning, such as role-plays, real-world experience, and ongoing supervision (Rowe et al., 2021, 2022).

## 5. Conclusions

This scalable, self-paced digital training program accessible via a smartphone was well received by ASHAs, who reported high satisfaction and described valuing the opportunity to learn how to recognize, refer, and support individuals living with schizophrenia. Notably, participants indicated that this was the first time they had heard about schizophrenia as a medical condition, highlighting the importance of increasing awareness and addressing misconceptions at the community level. However, this pilot study had several limitations, including the small sample size and absence of statistically significant improvements in knowledge scores, which limit conclusions about the training’s impact. Further research is needed to determine whether this training can improve ASHAs’ knowledge and whether these skills translate into practice and meaningful impact through earlier detection and initiation of care. Nonetheless, this study offers preliminary insights into the feasibility and acceptability of delivering digital schizophrenia training to ASHAs in rural India and represents an important early step toward future research aimed at strengthening integration of schizophrenia care into rural primary health systems.

## Figures and Tables

**Table 1:** Participant demographic characteristics.

Demographic Characteristic	Sample Size (Total N = 20)	
	N	%
**Age (in years)**		
26 to 30 years	5	25%
31 to 35 years	9	45%
36 to 40 years	4	20%
41 to 45 years	2	10%
**Type of Community Health Worker**		
ASHAs	17	85%
ASHA Facilitators	3	15%
**Education Level**		
8th to 10th grade	10	50%
10th to 12th grade	3	15%
Graduation	6	30%
Post-graduate level	1	5%
**Work Experience (in years)**		
4 to 9 years	9	45%
10 to 13 years	6	30%
14 and above	5	25%

**Table 2:** Participant satisfaction ratings with the digital training program.

Domains of Satisfaction	Responses from N = 20 Participants
	Mean (SD)
**Acceptability**	
I could find answers to questions I had about the coursework	5.45 (0.60)
Answers to questions about the coursework were easy to understand	5.4 (0.59)
The instructor in the recorded lecture cared about helping me to learn	5.45 (0.51)
The recorded lecture used a respectful tone in the recording	5.35 (0.48)
The recorded lecture used a friendly tone	5.2 (0.52)
The recorded lecture used familiar language and expressions	5.5 (0.51)
**Appropriateness**	
The coursework held my attention.	5.45 (0.51)
The instructional methods used in this course held my attention.	5.15 (1.18)
I enjoyed the instructional methods used in this course.	5.5 (0.51)
The instructional methods engaged me in the course.	4.7 (1.34)
I enjoyed completing the coursework.	5.45 (0.51)
The coursework was interesting to me.	5.3 (0.48)
**Adoption**	
In general, the coursework was useful to me.	5.35 (0.74)
The coursework was beneficial to me.	5.7 (0.47)
I found the coursework to be relevant to my future.	5.25 (0.96)
I will be able to use the knowledge I gained in this course.	5.55 (0.51)
The knowledge I gained in this course is important for my future.	5.4 (0.50)
**Feasibility**	
I had the opportunity to decide for myself how to meet the course goals.	5.05 (1.35)
I was confident that I could succeed in the coursework.	5.45 (0.51)
I had the freedom to complete the coursework my own way.	5.35 (0.48)
I felt that I could be successful in meeting the academic challenges in this course.	5.45 (0.60)
I had options in how to achieve the goals of the course.	5.2 (0.95)
I was capable of getting a high grade in this course.	5.2 (0.69)
I had control over how I learned the course content.	5.2 (0.95)
Throughout the course, I felt that I could be successful on the coursework.	5.35 (0.58)
I had flexibility in what I was allowed to do in this course.	5.4 (0.50)

**Table 3:** Summary of qualitative findings about acceptability of the digital training program.

Theme	Domain (Feasibility/Acceptability)	Key Insights	Representative Quotes
**Acceptability of the digital training**	Acceptability (content, format, usability)	Training content was clear, culturally appropriate, and easy to understand; videos and visuals enhanced learning; ability to revisit content increased perceived usefulness.	“*The language was good and easy to understand* … *it was just our mother tongue Hindi*.” (FGD1)
	Feasibility (platform navigation)	Most participants found the platform easy to use and valued flexibility, though some reported difficulty navigating questions or interactive elements.	“*We can watch it again and again if you are not able to understand*.” (FGD2)
	Acceptability (comparative value)	Digital training was viewed as more flexible than face-to-face training, allowing repeated review at one’s convenience.	“*On mobile, you can try it again and again* … *according to your convenience*.” (FGD1)
**Limited prior understanding and misconceptions about schizophrenia**	Acceptability (relevance, perceived need)	Participants had little or no prior knowledge of schizophrenia as a treatable mental health condition; symptoms were often attributed to witchcraft or possession.	“*I never thought that there is a disease with this name*.” (FGD3)
	Feasibility (contextual barriers)	Stigma, fear, and reliance on faith healers were common, limiting prior help-seeking and recognition of illness.	“*People say someone has done black magic . now after training I know it is a mental health disease*.” (FGD2)
**Knowledge and skills gained from the training**	Acceptability (perceived benefit)	Training improved understanding of symptoms, treatability, and referral pathways; participants valued their new role in educating families.	“*Now we know what schizophrenia is and that it can be treated*.” (FGD2)
	Feasibility (application in practice)	ASHAs felt more confident identifying symptoms, counseling families, and facilitating referrals to health centers.	“*We will explain to the family that it is not black magic* … *it is a disease*.” (FGD2)
	Acceptability (empowerment, motivation)	Participants expressed a sense of responsibility and purpose in guiding patients away from superstition toward treatment.	“*It is our job to show him the right path*.” (FGD1)
